# Ginseng for Liver Injury: Friend or Foe?

**DOI:** 10.3390/medicines3040033

**Published:** 2016-12-17

**Authors:** Tae-Woo Kim

**Affiliations:** Graduate School of Medicine, School of Medicine, CHA University, Seongnam-shi, Gyunggi-do 13488, Korea; k-taewoo@chauniv.ac.kr; Tel.: +82-10-2851-3947

**Keywords:** ginseng, *Panax* sp., hepatoprotective, hepatotoxic

## Abstract

*Panax* sp., including *Panax ginseng* Meyer, *Panax quiquifolius* L., or *Panax notoginseng* (Burk.) FH Chen, have been used as functional foods or for traditional Chinese medicine for diabetes, inflammation, stress, aging, hepatic injury, and cancer. In recent decades, a number of both in vitro and in vivo experiments as well as human studies have been conducted to investigate the efficacy and safety of various types of ginseng samples and their components. Of these, the hepatoprotective and hepatotoxic effects of ginseng and their ginsenosides and polysaccharides are reviewed and summarized.

## 1. Introduction

**Figure 1:**
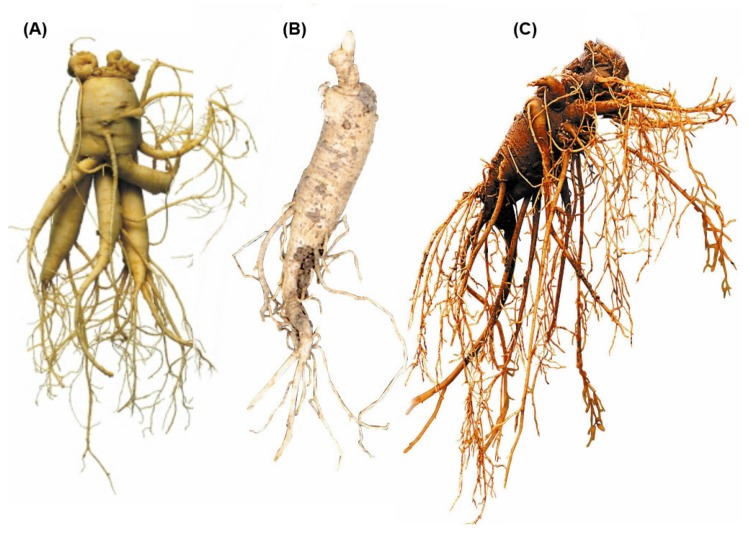
Photos of the roots of *Panax ginseng* Meyer (**A**); *Panax quiquifolius* L. (**B**); and *Panax notoginseng* (Burk.) FH Chen (**C**).

The term ginseng refers to the dried root of the *Panax* spp. (family Araliaceae), including *Panax ginseng* Meyer (PG, Asian Ginseng or Korean ginseng), *Panax quiquifolius* L. (PQ, American Ginseng), and *Panax notoginseng* (Burk.) FH Chen (PN; Figure 1) [1,2]. These have been used worldwide as herbal medicines or as functional foods. Of them, PG is the most frequently used. Shibata et al. isolated ginseng saponins from PG as bioactive, unique components in 1963, and named them ginsenosides [3,4]. Thereafter, other researchers have isolated approximately 200 components from PG, PQ, and PN, including other ginsenosides, polysaccharides, and polyacetylenes. 

The unique components of ginseng are chiefly ginsenosides, which are classified into dammarane-type, ocotillol-type, and oleanane-type oligoglycosides, as well as polysaccharides. Dammarane-type ginsenosides are further classified into two types: protopanaxadiol and protopanaxatriol [4,5]. Ginsengs and their main constituents (ginsenosides and polysaccharides) have been reported to exhibit many pharmacological properties: anti-oxidant, anti-aging, anti-inflammatory, memory-enhancing, anxiolytic, anti-diabetic, and hepatoprotective effects [2,5,6,7]. Most importantly, ginseng extracts have been shown to improve learning and memory in healthy, aged, or brain-damaged animals [8,9]. In particular, ginsenoside Rb1 and 20-*O*-(β-d-glucopyranosyl)-20(*S*)-protopanaxadiol (compound K, CK) were reported to be the major players in improving memory disorders, axonal atrophy, and synaptic loss in a mouse model of Alzheimer’s disease (AD) induced by an intracerebroventricular injection of amyloid β peptide (Aβ) (25–35) [10]. PG extract has also been found to restore glucose homeostasis and insulin sensitivity [11], and prevent type 2 diabetes mellitus and possibly obesity in mice through insulin resistance index improvement and diameter decrease in white and brown adipocytes [12]. With regards to diabetes mellitus, ginsenoside Rb2 may inhibit palmitate-induced gluconeogenesis [13,14]. Of the PG polysaccharides, ginsan has been shown to be a potent immunomodulatory agent inducing production of the cytokines: tumor necrosis factor (TNF), interleukin (IL)-1β, IL-2, IL-6, IL-12, interferon (IFN)-γ, and granulocyte-macrophage colony-stimulating factor [15]. Acidic polysaccharides exhibit strong immune-stimulating activity via stimulation of splenic T cell and B cell proliferation, increasing nitric oxide production from peritoneal macrophages, and enhancing natural killer cell activity [16]. Ginseng total saponin can modulate dopaminergic activity at both presynaptic and postsynaptic dopamine receptors [17]. Ginsenoside Rh2 and CK were found to ameliorate ischemic brain injury [18]. Ginsenosides have been demonstrated to lower plasma cholesterol and triglyceride levels, and inhibit aortic atheroma formation in an animal mode of high-cholesterol diet-induced hypercholesterolemia [19]. Ginsenoside Rb1 and CK have been reported to exhibit hepatoprotective effect against *tert*-butyl hydroperoxide-induced liver injury [20]. Red ginseng (RG, the steamed root of PG) was shown to attenuate ethanol-induced steatosis and oxidative stress [20]. Ginseng has been reported to have a strong anti-cancer effect against many different cancer types; ginsenosides, in particular, have been found to be cytotoxic against various cancer cells. Among them, CK most potently inhibits the growth of cancer cells [21], while PG as an adjuvant can enhance the anti-proliferative effect of chemotherapy [22]. As illustrated above, it is well known that the two key bioactive component groups of ginseng are ginsenosides and polysaccharides; however, the pharmacological effects of polysaccharides have not been studied nearly as extensively as those of ginsenosides.

Ginsengs (PG, PQ, and PN) and their components have been shown to have therapeutic effects against numerous hepatic injuries (Table 1). However, given that there are many bioactive components of ginseng, including the ones yet to be isolated with unknown biological effects, there has been concern regarding the potential adverse interaction between ginseng and other drugs. Thus, this review aims to describe the therapeutic effects of ginseng against many hepatic injuries along with the underlying mechanisms of such therapeutic effects. As the use of herbs including ginseng is increasing, the risk of liver toxicity is also increasing [23]. Therefore, this review will also discuss the potential hepatotoxicity of ginseng to better understand the risk ginseng use poses to the liver. 

## 2. Multiple Efficacies for Hepatic Injuries

### 2.1. Ethanol-Induced Hepatic Injury

The pathogenesis of ethanol-induced hepatic injury involves three stages: ethanol-induced (alcoholic) fatty liver, alcoholic hepatitis, and alcoholic cirrhosis. Alcoholic fatty liver is thus the preceding condition to the more severe pathologies. It is therefore critical to reverse ethanol-induced hepatic injury at the alcoholic fatty liver stage. RG extract improves liver function by attenuating ethanol-induced steatosis and oxidative stress [24]. Chronic ethanol-induced hepatic injury condition leads to elevations in serum aspartate transaminase (AST) and alanine transaminase (ALT). However, pretreatment with RG extract maintained serum ALT activity and malondialdehyde (MDA) concentration within the normal range after short-term ethanol ingestion [25]. Of the constituents responsible for the hepatoprotective effect of RG, Rg3 and Rh2 were found to be the primary ginsenosides of RG that produced medicinal effects against ethanol-induced oxidative injury [26]. The second stage of ethanol-induced hepatic injury is alcoholic hepatitis. Given that the next stage is liver cirrhosis, it is imperative to reverse alcoholic hepatitis to prevent further pathologic progression. PN attenuates the rise in serum AST and ALT due to chronic ethanol-induced hepatotoxicity [27]. More specifically, PN reduces liver ALT and AST levels, collagen fiber deposition, and transforming growth factor (TGF)-β1 expression that are normally observed with the increase in the expression quantity of SMA/MAD homology protein 7 (Smad7) in rats with ethanol-induced liver injury [28]. In particular, ginsenoside Rg1 isolated from PN has been found to suppress ethanol-induced elevations in blood AST and ALT, and TNF in rats with alcoholic hepatitis, which significantly decreases pathological injury as well as improving liver function [29]. These findings suggest that the ameliorating effects of ginsengs, including PG and PN, against ethanol-induced hepatitis may be attributed to their constituents, i.e. such as ginsenosides Rg1, Rg3, and Rh2, which potently inhibit reactive oxygen species (ROS) production and collagen deposition in the blood and liver.

### 2.2. Lipopolysaccharide-Induced Hepatic Injury

Gram-negative bacterial infection is one of many causes of hepatic injury. Lipopolysaccharide (LPS) is critically responsible for this type of hepatic injury. Lipopolysaccharide is a well-known bacterial endotoxin of gram-negative bacteria. RG suppresses alcohol-induced TNF levels in mice, along with the increase in IL-10 level [30]. PN also markedly attenuates the elevated serum AST and ALT levels induced by LPS-derived hepatic injury in rats [31]. Heat-processed PG (sun ginseng, SG) protects against LPS-induced rat liver injury by reducing the elevated level of thiobarbituric acid reactive substance (TBARS), activation of nuclear factor (NF)-κB, and expression of inducible nitric oxide synthase (iNOS), in addition to upregulating heme oxygenase (HO)-1 expression [32,33]. Ginsenoside Rc inhibits TNF-α and IFN-γ expression, and attenuates activator protein 1 (AP-1) activation in rats with LPS/D-galactosamine-induced hepatitis [34]. Ginsenoside Rd inhibits iNOS and cyclooxygenase (COX)-2 expression and NF-κB activation in the liver of rats treated with LPS [35]. In addition to ginsenosides Rc and Rd, ginsenosides Re and Rg1 were found to reduce blood ALT in mice with LPS/D-galactosamine-induced liver injury [36]. Ginsenoside Rg3 has also been reported to significantly decrease LPS-induced blood AST and ALT levels and liver HO-1 expression in rats [37]. These findings suggest that ginseng, including PG and PN, and their constituents, such as ginsenosides Rc, Rd, and Rg3, attenuate LPS-induced hepatitis by inhibiting iNOS and COX-2 via the inhibition of NF-κB activation and upregulating of HO expression. 

### 2.3. Hepatitis Virus-Induced Hepatic Injury

There are several types of hepatitis viruses, A, B, C, D, and E, all five of which can cause acute hepatitis. Two of the five types, hepatitis virus B and hepatitis virus C, can cause chronic hepatitis leading to liver cirrhosis. Liver cirrhosis is a state requiring close monitoring, as it is the state preceding liver carcinoma. RG and its constituents, ginsenosides Rg1 and Rb1, have been shown to exhibit a hepatoprotective effect against hepatitis virus A by decreasing hepatitis virus titers [38]. Drugs for chronic hepatitis virus C have been developed but pose a practical problem owing to their cost, as the full drug regimen for hepatitis virus C is extremely expensive. Under these circumstances, ginseng may play a critical role through the prevention of permanent infection by the previously mentioned hepatitis viruses including hepatitis virus C. Although sufficient studies have not yet performed, the published reports suggest that RG and its constituents, ginsenosides Rg1 and Rb1, may attenuate hepatitis induced by hepatitis B and C viruses. However, the mechanism by which this occurs should be fully elucidated.

### 2.4. Carbon Tetrachloride-Induced Hepatic Injury

Formerly used as a precursor to refrigerants, carbon tetrachloride (CCl_4_) is a hepatotoxin [108]. It is activated by cytochrome p450 (CYPs), and possibly CYP3A, to form the trichloromethyl radical, CCl_3_*. This radical binds to cellular molecules such as nucleic acids, proteins, and lipids, impairing crucial cellular processes. In fact, the CCl_4_-induced hepatic injury model has been widely used to assess the medicinal effect of potential hepatoprotective agents. 

PG restores liver superoxide dismutase (SOD) and catalase activities, and maintains physiologic biochemical parameters, and histological structures in rats treated with CCl_4_ [39]. Specifically, PG protects rats from ALT, AST, alkaline phosphatase (ALP), and liver peroxide elevation induced by CCl_4_ [40]. PG pretreatment significantly decreases the cluster of differentiation 68 (CD68+) staining area and fiber accumulation in the liver of rats with CCl_4_-induced acute hepatotoxicity [41,109]. RG partially restores CCl_4_-induced serum AST and ALT levels [43]. It also notably down-regulates CCl_4_- or TGF-β1-induced fibrogenic genes such as plasminogen activator inhibitor I in human hepatic stellate cell lines [42]. The RG saponin fraction not only partially lower elevated serum ALT and AST, but also histologically reverses liver vacuolization and lymphoid cell aggregation in rats treated with CCl_4_ [44]. RG saponin inhibits P450-associated monooxygenase activities in a dose-dependent manner [45]. In particular, the inhibitory effects of RG saponin on P450 enzymes may have a critical role in CCl_4_-induced lipid peroxidation in rat liver microsomes [45]. 

PN significantly inhibits CCl_4_-induced liver injury in rats, with a methanol extract being more effective than a water extract [46]. The PN saponin fraction effectively decreases the levels of IL-1, IL-6, NF-κB, TNF, and TGF-β, and increases the level of IL-10 [47]. The PN saponin fraction further up-regulates matrix metalloproteinase (MMP)-3 expression, as well as down-regulating the tissue inhibitor of metalloproteinase (TIMP)-1 expression, leading to improvement in collagen degradation. Ginsenoside Ro, an oleanane-type saponin, inhibits the increase in serum ALT and AST levels in rats with CCl_4_-induced acute hepatitis [49]. The PN saponin fraction also decreases body and hepatic fat deposition, plasma and hepatic triglycerides, hepatic prostaglandin E2, hydroxyproline, and TIMP-1 levels [48]. Ginsenoside Rb1 further inhibits CCl_4_-induced protein (34, 118, 63 kDa) phosphorylation by modulating Ca^2+^/calmodulin-dependent protein kinase in rats [50]. Ginsenoside Rg1 exerts protective effects in a rat model of CCl_4_-induced hepatic fibrosis via promotion of the nuclear translocation of nuclear factor E2-related factor 2 (Nrf2) and expression of antioxidant enzymes such as SOD, glutathione peroxidase (GSH-Px), and catalase [51]. 

Treatment with ginsan, a polysaccharide extracted from PG, markedly suppresses CCl_4_-induced serum ALT and AST levels and hepatic histological necrosis in rats [52]. Induction of anti-oxidant protein contents, such as SOD, catalase, and GSH-Px, as well as restoration of the hepatic glutathione (GSH) concentration by ginsan form the foundation of this hepatoprotective effect. [52]. 

Overall, CCl_4_ produces radicals and causes liver injury, and the representative constituents of ginseng, ginsenosides and polysaccharides, attenuated CCl_4_-induced hepatitis in vitro and in vivo. These ameliorating effects may be dependent on scavenging ROS through the regulation of SOD and catalase expression, and inhibition of NF-κB activation. 

### 2.5. Hydroperoxide-Induced Hepatic Injury

The production of reactive oxygen intermediates by oxidants results in liver cell injury [54]. Hydrogen peroxide-mediated oxidant stress may induce necrosis and apoptosis, leading to cell death. PG extract increases the expression of hydrogen peroxide-induced oxidative stress signals, lipid peroxidation and oxidative DNA damage [63,110]. PG significantly decreased aflatoxin-induced lipid peroxidation and expression of fatty-acid synthesis enzymes in the liver of rats [63]. PG extract restores the such as c-Jun-*N*-terminal kinase and stress-activated protein kinase expression, increases the expression of mitochondrial cytochrome c released caspase-3 activation, and reduces malondialdehyde (MDA) production [55]. Fermented black ginseng induced both the expression and activity of antioxidant enzymes, such as SOD, catalase, and GSH-Px in hydrogen peroxide-stimulated HepG2 cells [53]. Many components of ginseng have been shown to exhibit hepatoprotective effects against such oxidative stress. Of these components, ginsenoside Rb1 exerted an antifibrotic effect on hydrogen peroxide-stimulated HSC-T6 cells by inhibiting the activation, proliferation, and expression of collagen, TGF-β1, and MMP-2 [56]. Ginsenoside Rb2 restores gap junctional intercellular communication in rat liver epithelial cells otherwise injured by hydrogen peroxide [58]. Ginsenosides Rb1, CK, Rg3, and Rh2 are reported to the major contributors to PG’s hepatoprotective effect against tert-butylhydroperoxide-induced hepatic injury [20,57]. 

Hydrogen peroxide and tert-butyl hydroperoxide produce ROS and cause hepatitis. PG and its ginsenosides CK and Rh2 attenuate hydroperoxide-induced hepatitis by scavenging ROS. 

### 2.6. Radiation-Induced Hepatic Injury

The frequency of the electromagnetic radiation produced by cell phones can induce oxidative injury and corresponding morphology change in the liver [111]. PG ethanol extract reverses the levels of radiation-depleted glutathione and antioxidant enzymes (SOD, catalase, and glutathione *S*-transferase) as well as the elevation in lipid peroxidation (LPO) levels in the blood and liver [59,60]. The total saponin fraction of the PG ethanol extract also restores the radiation-impaired expression of CYP 1A2, 2B1, 2E1, and 3A4 in rats [61]. PQ clearly decreased the contents of liver MDA and Nrf2 protein expression (*p* < 0.05), and clearly increased the contents of liver SOD, gutathione, and GSH-Px in the rats induced by 900 MHz cell phone [62]. These findings suggest that ginsengs, including PQ and RG, may attenuate radiation-induced hepatitis by scavenging ROS via the regulation of SOD expression in the liver.

### 2.7. Aflatoxin-Induced Hepatic Injury

Aflatoxins, food-borne mycotoxins, are carcinogens that can induce liver carcinoma through levels of reduced glutathione, SOD, catalase, vitamin C, and total proteins, with a significant reduction in lipid peroxidation levels in the liver of 7,12-dimethylbenz[a]anthracene-croton oil-treated mice [64]. RG decreased the elevated AST, ALT, and MDA levels, and increased SOD, catalase, and GSH-Px activities in an aflatoxin B1-induced rat model [63]. RG extract also restores blood biochemical parameters such AST, ALT, and MDA, as well as liver histological parameters, in rats injured with simultaneous stimulation by aflatoxin B1 and fumonisin [64,65]. Furthermore, prolonged administration of RG extract significantly inhibits hepatoma and pulmonary tumor proliferation induced by aflatoxin B1 and urethane in mice [66,67,68]. Aflatoxins are biotransformed into their carcinogenic metabolites such as aflatoxin B1-exo-8,9-epoxide in the liver. However, ginsengs suppress aflatoxin-induced ROS production and increase SOD expression. These findings suggest that PG and RG may attenuate aflatoxin-induced hepatitis by suppressing ROS production through the regulation of SOD expression in the liver.

### 2.8. Benzo[a]pyrene-Induced Hepatic Injury

Benzo[a]pyrene is a carcinogen that interferes with transcription via through DNA intercalation [69]. Wild PG exerts its hepatoprotective effect by attenuating the increased serum AST and ALT levels from benzo[a]pyrene-induced hepatic injury [69]. Specifically, ginsenoside Rg3 prevents benzo[a]pyrene-induced DNA damage [70]; ginsenosides CK, O, and Mc1 inhibit benzo[a]pyrene-induced mutagenicity, and ginsenosides CK and Mc1 reduce the frequency of chromosome aberration induced by benzo[a]pyrene [71]. These findings suggest that ginseng containing the ginsenosides Rg3 and CK may attenuate benzo[a]pyrene-induced hepatitis by suppressing benzo[a]pyrene-induced mutagenicity through the inhibition of ROS production.

### 2.9. Cadmium-Induced Hepatic Injury

Cadmium (e.g., CdCl_2_) is pervasive in our environment because of its widespread use in many industries, despite its severe hepatotoxicity. PG and RG attenuate cadmium-induced hepatotoxicity; they have been found to lower the elevated AST, ALT, and lactate dehydrogenase (LDH) levels in rats [72,75]. PG also improves cadmium-suppressed body weight gain, and restores the increase in TBARS and decrease in GSH concentrations in the liver of chicks after cadmium treatment [73]. Furthermore, PG decreases CdCl_2_-induced LPO, ALT, and AST levels, and increases CdCl_2_-suppressed serum glutathione levels and alkaline phosphatase activity [74]. PN also ameliorates cadmium-induced liver injury in mice [76]. These findings suggest that ginsengs, including PG, RG, and PN, may attenuate cadmium-induced hepatitis by scavenging ROS.

### 2.10. Hepatocellular Carcinoma

Hepatocellular carcinoma (HCC) is the most common cause of cancer-related death. Most cases of HCC develop from chronic liver diseases such as hepatitis B virus infection, hepatitis C virus infection, alcohol consumption, or non-alcoholic steatohepatitis, and are sometimes diagnosed only after progression to severe HCC [37]. Given that currently the most effective treatment for liver carcinoma is systemic chemotherapy, local ablation, partial liver resection, or transplantation, it is critical to practice preventive medicine to protect one’s liver, including vaccinations for hepatitis viruses and avoiding hepatotoxic agents.

PG pretreatment protects against the development of liver cancer induced by diethylnitrosamine (DEN) in rats [96], and has shown a cancer-preventive effect in patients with chronic hepatitis C infection [107]. Furthermore, PG decreases glutathione *S*-transferase P form (GST-P)-positive foci, which is a stable marker for rat hepatocarcinogenesis, in DEN-injected rats; it also down-regulates the expression of cyclin D1, cyclin G1, cell division cycle 2a (Cdc2a), and insulin-like growth factor-1 (Igf-1), which are involved in the p53 signaling pathway [97]. RG exhibits an anticarcinogenic effect on the development of DEN-induced liver cancer in rats [83]. RG promotes tumor necrosis factor-related apoptosis-inducing ligand (TRAIL)-derived apoptosis in HepG2, Huh-7, and Hep3B cell lines via up-regulation of C/EBP homologous protein [84]. In extensive preclinical and epidemiological studies, RG has been shown to have cancer-preventive effects on many types of cancers, not just liver cancer [85,86]. Ginsenosides Rg3 and Rh2 exhibit a potent cytotoxicity effect on the human hepatoma cell line, HepG2, by stimulating p53-mediated cell cycle arrest at the G1 to S phase transition, leading to apoptosis through the caspase signaling pathway [87]. Ginsenoside Rs4 induces apoptosis, the effect of which is closely related to the downregulation of both cyclin E- and A-dependent kinase activity as a consequence of selectively elevating the protein levels of p53 and cyclin-dependent kinase inhibitor 1 (p21^WAF1^) in hepatoma SK-HEP-1 cells [88]. Ginsenoside Rh2 induces apoptosis by increasing the proteolysis of cyclin-dependent kinase inhibitor p21WAF1/CIP1, an inhibitor of cyclin kinases in SK-HEP-1 cells [89]. Ginsenoside Rk1 inhibits telomerase activity in human hepatocellular carcinoma HepG2 cells in vitro [90]. Ginsenoside Rk1 also induces apoptosis through the activation of caspases-8 and -3 [90]. Ginsenoside Rg3 exhibits the potent cytotoxicity in combined therapy with TRAIL [91]. Ginsenoside Rg3 also inhibits late stage autophagy-induced doxorubicin in HCC cells [92]. Furthermore, treatment with ginsenoside Rg3 in the presence of doxorubicin synergistically killed HCC cells and inhibited tumor growth in HCC xenografts in vivo. Ginsenosides Rg3 and Rh2 promote the uptake of MMC into tumor cells [93]. RG containing ginsenosides Rg3, Rg5, and Rh2 (all of which exhibit cytotoxic effects against tumor cells) as their main constituents prevent the occurrence of non-organ specific tumors [94]. RG and ginsenosides Rg3 and Rh2 have obvious cytotoxic and apoptotic effects in Hep3B cells via the mitochondria-mediated apoptosis pathway, which stimulates the release of mitochondrial cytochrome c, and activation of caspase-3 and Bax protein [95].

CK treatment of MHCC97-H cells decreases mitochondrial membrane potential (Δψm) and DNA damage [98]. CK significantly inhibits proliferation of and induces apoptosis in MHCC97-H cells through the Fas (CD95)- and mitochondria-mediated caspase-dependent pathways. CK inhibits growth of human hepatocellular carcinoma SMMC7721 cells through cell cycle arrest in the G0-G1 phase of the cell cycle. Specifically, CK up-regulates cytochrome c, p53, and Bax expression, and down-regulates pro-caspase-3 and pro-caspase-9 expression [99]. Exposure to CK leads to both apoptotic cell death and G1 arrest in Hep3B cells, but only results in G1 arrest in MDA-MB-231 and MKN28 cells [100]. CK up-regulates COX-2 expression and prostaglandin E2 production in MDA-MB-231 cells but not Hep3B cells, and induces sustained extracellular signal-regulated kinzse (ERK) activation; ERK inhibition was found to block CK-induced COX-2 expression and led to apoptosis. CK induces G1 arrest through the increase of p27Kip1 expression, followed by the decrease in CDK2 kinase activity [100]. Thus, CK induces both G1 arrest and COX-2-induced apoptosis. CK induces apoptosis in human hepatoblastoma HepG2 cells through the activation of procaspase-3 and 8. CK triggers the cleavage of cytosolic factors Bid and Bax and translocation of truncated Bid (tBid) into mitochondria [101]. Immunohistochemical staining revealed that Bid expression in subcutaneous tumor and liver metastatic tissues decreased dramatically in CK-treated mice compared to untreated controls, which also implies that Bid may play a critical role in the growth and progression of HCC [102]. CK strongly attenuates colony formation, adhesion, and invasion of HCC cells in vitro, and dramatically inhibits spontaneous HCC metastatic growth in vivo through the inhibition of the NF-κB pathway [103]. Furthermore, CK inhibits metastasis by inhibiting MMP2/9 expression through NF-κB activation. Panaxadiol-treatment induces cell death-dependent activation of Cdk2 kinase activity, which is functionally associated with depolarization of the mitochondrial membrane potential and subsequent cytochrome c release [104]. 25-Methoxydammarane-3,12,20-triol (25-OCH(3)-PPD) isolated from PN increases the levels of cleaved caspase-3, and decreases the ratio of Bcl-2/Bax and the expression of survivin via c-FLIP (a master of anti-apoptotic regulator)-mediated NF-κB activation [105]. A polysaccharide from PN inhibits the growth of H22 liver cancer cells, and significantly prolongs the survival of tumor-bearing mice via the induction of activated CD4+ T-cells and serum IL-2 [106]. 

Ginseng and its ginsenosides attenuate HCC developed as a result of from hepatitis B and C virus infection, excessive and chronic alcohol consumption, and chemical exposure by inducing apoptosis via the cycle arrest; they suppress metastasis by inhibiting MMP expression through the regulation of NF-κB activation. Furthermore, in combination with an autophagy-inducer doxorubicin, a well-known anti-cancer drug, they synergistically induce apoptosis in HCC.

### 2.11. Liver Regeneration and Transplantation

Because the liver has powerful regenerative abilities, the most effective therapeutic approach to a single, small liver tumor is partial hepatectomy. However, even though regenerative ability of the liver is high, measures for the promotion of liver regeneration is needed for fast recovery without further complications. RG has promoting effect on liver regeneration after partial hepatectomy [77]. RG has a liver regeneration effect even in rats with 70% hepatectomy [78]. RG accelerates liver regeneration, and ameliorates liver injury after hepatectomy in dogs [77]. RG increases liver weight and hepatocyte proliferation in rats after hepatectomy [78]. RG restores AST and ALT levels and the number and area of lipid droplets. 

The final treatment option for either liver cirrhosis or liver carcinoma is liver transplant. The major cause for the primary graft dysfunction is ischemia-reperfusion induced hepatic injury [112]. Measures to overcome ischemia/reperfusion-induced hepatic injury are needed for successful liver transplant. The PG saponin fraction attenuates ischemic reperfusion injury in experimental obstructive jaundice rats; it was found to reduce ALT, AST, and MDA levels [79]. PN saponin preconditioning protects liver grafts in rats from ischemia-reperfusion injury through the ananti-apoptotic pathway [80]. PN saponins exhibit antiapoptotic effects by inhibiting the expression of TNF and caspase-3 and enhancing the expression of B cell lymphoma 2 (Bcl-2). Additionally, these PN saponins protect hepatocytes against ischemic reperfusion injury in the early stages of transplantation [80]. PN saponins have been found to attenuate gut ischemia/reperfusion-induced hepatic microvascular dysfunction and inflammatory responses in the early phase via enhancement of NO production, as well as sequential hepatocellular damage via an anti-inflammatory effect [81]. With anti-inflammatory and anti-apoptotic properties, ginsenoside Rg1 exerts a hepatoprotective effect against ischemia-reperfusion injury [113]. Ginsenoside Rg1 also inhibits the inflammatory response through the NF-κB signaling pathway [114]. 

These results suggest that PG and PN and their ginsenoside Rg1 attenuate ischemic reperfusion or hepatectomy-induced hepatitis by inhibiting NF-κB activation. 

## 3. Combination Therapy

### 3.1. Additive or Synergistic Effect

When two hepatoprotective agents are used together, they may produce an additive or synergistic effect. Standardized ginseng extracts along with trace elements and multivitamins exert a protective effect against hepatotoxin-induced chronic liver disease in the elderly [115]. When used with dietary honey, ginseng exhibits a greater hepatoprotective effect against CCl_4_-induced hepatic injury [116]. Recently, herbal medicines, including the root of Polygonum multiflorum, have been recognized as the common cause of drug-induced liver injury [23,117,118,119,120]. Ginseng is the representative ingredient in the herbal formulae. Therefore, the simultaneous use of ginseng with other drugs (herbal or western medicines) should be considered carefully in the clinic.

### 3.2. Acetaminophen

As an over-the-counter drug, acetaminophen has widely been used to treat many conditions: headache, muscle ache, toothache, and fever. Even though it is an over-the-counter drug, one has to pay close attention to hepatotoxicity at high doses. The most common cause of hepatitis is drugs, including acetaminophen and herbs, followed by viruses and others [114,121,122,123]. Fermented ginseng and its major constituent compound K attenuate acetaminophen-induced hepatic injury [123]. Panaxatriol saponin ameliorates liver injury induced by acetaminophen via restoration of thioredoxin-1 and pro-caspase-12 [114]. *N*-acetyl-*p*-benzoquinone is a toxic metabolic intermediate of acetaminophen [124]. Cytochrome P450 2E1 metabolizes acetaminophen into *N*-acetyl-*p*-benzoquinone [125]. Ginsenoside Rg3 exhibits its hepatoprotective effect on acetaminophen-induced hepatic injury by inhibiting cytochrome P450 2E1 so that a toxic intermediate cannot be produced [126]. These results suggest that ginsengs may improve acetaminophen-induced liver injury rather than damage the liver.

## 4. Adverse Interactions

It is important to understand drug–drug interactions to avoid adverse drug interactions; similarly, one also needs to have a clear understanding of dietary supplement-to-medication interactions to avoid adverse interactions. According to case reports, ginseng may inhibit the analgesic effect of opioids [126]. Cytochrome P450 enzymes are responsible for the metabolism of many medications. Given that ginseng inhibits the CYP3A4 enzyme that metabolizes imatinib, the use of imatinib along with ginseng can result in hepatic damage due to improper imatinib metabolism and corresponding excretion [127]. CYP3A4 is also responsible for raltegravir metabolism in the liver [128]. There has been is a case report in which the use of raltegravir with PG ultimately produced hepatic damage owing to CYP3A4 inhibition by PG [128]. Ginsenosides, particularly 20(*S*)-protopanaxadiols, are potent inhibitors for organic anion-transporting polypeptide 1B3, which is associated with pharmacokinetics of various drugs [129]. 

Oral administration of RG to male and female rats at dose levels of 0, 500, 1000, and 2000 mg/kg/day for four weeks did not lead to death or clinical symptoms [130]. More specifically, no abnormalities in body weight, food consumption, ophthalmology, urinalysis, hematologic parameters, serum biochemistry, gross findings, organ weights, or histopathological parameters were observed. Therefore, the no observed adverse effect level (NOAEL) of RG extract was established to be 2000 mg/kg/day. In addition, no mortality and treatment-related toxicity effects were observed upon the administration of 25-OCH3-PPD to rats at any dose level (150, 300 and 600 mg/kg) for 92 consecutive days [131]. Oral administration of Rg1 does not induce significant abnormality in the brain, heart, kidney, liver, and lung structure at any dose based on hematoxylin and eosin staining [132]. Even when Rg1 was mixed with salvianolic acid B, no significant toxicity was detected [132]. Many studies have reported that ginseng combined with or without drugs might ameliorate various diseases including acute liver disease; however, some caution must be exercised when combining ginseng with drugs. The use of ginseng in combination with herbs should also be undertaken carefully in the clinic.

## 5. Conclusions

**Figure 2:**
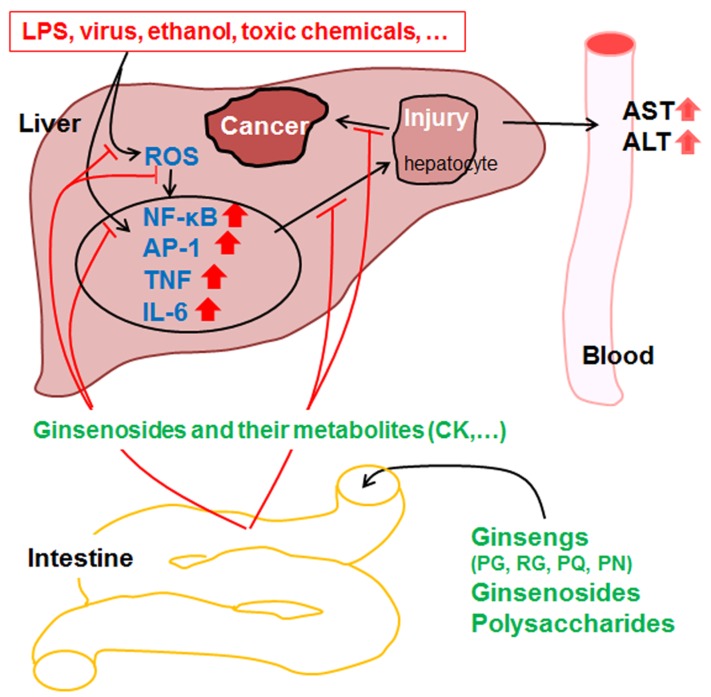
The hepatoprotective mechanism of ginsengs and their constituents proposed in in vitro and in vivo experiments. AP-1, activator protein 1; AST, aspartate transaminase; ALT, alanine transaminase; CK, compound K; LPS, lipopolysaccharide; , IL-6, interleukin 6; NF-κB, nuclear factor kappaB; PG, *Panax ginseng* Meyer; PN, *Panax notoginseng* (Burk.) FH Chen; PQ, *Panax quiquifolius* L.; and ROS, reactive oxygen species; TNF, tumor necrosis factor.

Ginseng extract and its saponins exhibit hepatoprotective effects against various hepatic injuries caused by chemical substances and hepatitis viruses. These causes of hepatitis produce ROS and activate inflammation signaling pathways such as the NF-κB pathway. Ginseng potently inhibits ROS production and the inflammation-signaling pathway. Moreover, as unique constituents of ginseng, ginsenosides have been found to inhibit liver carcinoma proliferation, promote liver regeneration, and prevent liver ischemia through anti-oxidative, anti-inflammatory, and anti-apoptotic mechanisms (Figure 2). Moreover, when used with vitamin or acetaminophen, the ginseng components were observed to exert additive or synergistic effects on hepatic injuries. However, these ginseng components inhibit CYP3A4, leading to serious hepatotoxicity when used with imatinib or raltegravir. In this respect, when a ginseng extract or its component is used clinically along with other drugs, the extract or component needs to be examined carefully to determine if it inhibits a particular CYP450 enzyme responsible for drug metabolism, as it might lead to increased efficacy and ultimately serious hepatotoxicity. 

## Figures and Tables

**Table 1:** Ginseng species and their constituents used in the hepatoprotective experiments.

Cause of Liver Failure	Tested Ginseng		Evaluated Parameter	Reference
	Species	Constituent		
Ethanol	RG	Rg3, Rh2	AST, ALT, MDA	[24,25,26]
	PN	Rg1	AST, ALT, collagen, TGF-β1, Smad	[27,28,29]
LPS	RG		TNF-α, IL-10	[30]
	PN		AST, ALT	[31]
	SG		MDA, NF-κB, iNOS, HO-1	[32,33]
	Rc		TNF-α, AP1	[34]
	Rd		iNOS, COX-2, NF-κB	[35]
	Re, Rg1		GPT	[36]
	Rg3		AST, ALT, HO-1	[37]
Virus (HAV)	RG	Rb1, Rg1	HAV titer	[38]
CCl_4_	PG		ALT, AST, LPO, ALP, CD68+	[39,40,41,42]
	RG	Saponin fraction	ALT, AST, liver vacuolation, fibrigenic genes, lymphoid cell aggregation, monooxygenase, LPO	[43,44,45]
	PN		ALT, AST	[46,47]
		Saponin fraction	IL-1, IL-6, NF-κB, TNF-α, and TGF-β, MMP-3, TIMP-1, PGE2, hydroxyproline, IL-10	[48]
		Ro	ALT, AST	[49]
		Rb1	Ca^2+^/calmodulin-dependent protein kinase	[50]
		Rg1	Nrf2. SOD, GSH-Px, catalase	[51]
	PG	polysaccharide	AST, ALT, SOD, catalase, GSH-Px	[52]
Hydroperoxide	BG		SOD, catalase, GSH-Px	[53]
		Rb1	proliferation, collagen	[54,55]
	PG	Rb1, CK	In vitro	[56]
		Rg3, Rh2	In vivo	[20,57]
		Rb2	Gap tight junction protein	[58]
Radiation	PG		GSH, SOD, catalase, LPO glutathione-*S*-transferase	[59,60]
		saponin	Cytochrome P450, MDA, Nrf2	[61]
	PQ		MDA, Nrf2, SOD	[62]
Aflatoxins	PG		LPO, fatty acid synthesis, GSH, SOD, catalase, ALT, AST, MDA	[63,64]
	RG		LPO, fatty-acid synthesis enzymes	[65,66,67,68]
Benzo[a]pyrene	PG		AST, ALT	[69]
		Rg3, CK, Mc1	DNA damage, chromosome aberration	[70,71]
Cadmium	PG		ALT, AST, LDH, GSH, MDA	[72,73,74]
	RG		ALT, AST, MDA	[75]
	PN		ALT, AST, GSH, MDA	[76]
Hepatectomy	RG		Liver regeneration, ALT, AST	[77,78]
Ischemia/reperfusion	PG		ALT, AST, MDH	[79]
		saponin	ALT, AST	[79]
	PN	Saponin	TNF-α, Caspase-3, Bcl-2, NO	[80,81]
		Rg1	NF-κB	[82]
Hepatocelluar carcinoma (in vitro)	RG		TRAIL-induced apoptosis, C/EBP	[83,84,85,86]
		Rg3, Rh2, Rs4,	cell cycle arrest, p53, p21WAF1	[87,88,89]
		Rk1	Caspase-3, -8	[90]
		Rg3	Autophagy, T cell (with, TRAIL MMC, or doxorubicin)	[91,92,93]
		Rg5, Rg3, Rh2	mitochondrial cytochrome c, caspase-3, Bax	[94,95]
Hepatocelluar carcinoma (in animal)	PG (DEN)		PST-P, cyclins	[96,97]
	RG (DEN)		DNA, RNA, glycogen, γ-GPT, SDH, 5′-NT	[97]
	CK		mitochondrial membrane potential, DNA damage, Fas, caspase, cell cycle arrest, cytochrome c, p53, Bax, PGE2, ERK, procapase-3, -8, Bid, NF-κB	[98,99,100,101,102,103,104]
	25-OCH(3)-PPD		caspase-3, Bcl-2, Bax, survivin	[105]
	polysaccharides		CD4(+) T, sIL-2	[106]
Hepatocelluar carcinoma (in human)	PG	HCV		[107]
	RG (Rg3, Rh2, and Rg5-rich)			[94]

ALP, alkaline phosphatase; ALT, alanine aminotransferase; AST, aspartate aminotranferase; BG, fermented black ginseng; CD68, cluster of differentiation 68; C/EBP, CCAAT-enhancer-binding protein; CK, compound K; COX, cyclooxygenase; DEN, diethylnitrosamine; GPT, serum glutamic-pyruvic transaminase; GSH, glutathione; GSH-Px, glutathione peroxidase; HAV, hepatitis A virus; HCV, hepatitis C virus; HO, heme oxygenase iNOS, inducible NO synthase; IL, interleukin; LPO, lipid peroxidation; MDA, malondialdehyde; MMC, mitomycin; MMP, matrix metalloproteinase; NF-κB, nuclear factor kappaB; NO, nitric oxide; Nrf2, nuclear factor E2-related factor 2; PG, *Panax ginseng*; PN, *Panax notoginseng*; PGE2, prostaglandin E2; PPD, protopanaxdiol; PQ, *Panax quiquifolius*; RG, red ginseng (steamed root of PG); Rg3, ginsengoside Rg3; sIL, secreted interleukin; Smad, SMA/MAD homology; SOD, superoxide dismutase; TNF, tumor necrosis factor; TGF, transforming growth factor; TIMP, tissue inhibitors of metalloproteinase; TRAIL, tumor necrosis factor-related apoptosis-inducing ligand.
